# High prevalence of HIV/AIDS risky sexual behaviors among street youth in gondar town: a community based cross sectional study

**DOI:** 10.1186/1756-0500-6-234

**Published:** 2013-06-15

**Authors:** Negash Tadesse, Tadesse Awoke Ayele, Zelalem Birhanu Mengesha, Kefyalew Addis Alene

**Affiliations:** 1 Family Health International Ethiopia, Addis Ababa, Ethiopia; 2 Department of Epidemiology and Biostatistics, Institute of Public Health, College of Medicine and Health Sciences, University of Gondar, Gondar, Ethiopia; 3 Department of Reproductive Health and Health Education, Institute of Public Health, College of Medicine and Health Sciences, University of Gondar, Gondar, Ethiopia; 4 Department of Public Health Officer, Institute of Public health, College of Medicine and Health Sciences, University of Gondar, Gondar, Ethiopia

**Keywords:** HIV/AIDS, Risky sexual behaviors, Street youth, Gondar

## Abstract

**Background:**

Street youth are vulnerable to HIV/AIDS and all kinds of health risks. This study assessed HIV/AIDS risky sexual behaviors and its predictors among street youth in Gondar town, Northwest Ethiopia.

**Methods:**

A community based cross sectional study was conducted on 467 street youth living in Gondar town. A pre tested and structured questionnaire via interview was used to collect data. Logistic regression analysis was used to identify predictors. Odds ratio with 95% CI was computed to assess the strength of associations.

**Results:**

A total of 288 (61.7%) respondents had sexual intercourse in their life time. Among these 264 (91.7%) had more than one lifetime sexual partners. In addition, 80.5% of them used condom inconsistently in the last 12 months. Khat chewing was found to be predictor of having multiple sexual partners. Rural former residence and longer duration of stay on the street are also identified as predictors of inconsistent condom use.

**Conclusion:**

High prevalence of HIV/AIDS risky sexual behaviors were observed among street youth in Gondar town. Interventions aimed at reducing sexual risky behaviors among street youth should focus on reducing the duration of stay on the street and chat chewing.

## Background

Sub-Saharan Africa including Ethiopia has been highly affected by HIV and currently accounts for more than two thirds of recent HIV infections worldwide [1]. The overall adult HIV prevalence in Ethiopia has remained almost similar in the Ethiopia Demography and Health Survey (EDHS) 2005 and 2011 which was 1.4% and 1.5% respectively. The prevalence is higher in urban areas (4.2%), where the street youth are living, than in rural areas (0.6%). It is relatively high among young respondents who report two or more sexual partners (6.7%) in the 12 months before the survey and among those who used a condom inconsistently (2.4%) [2,3]. This high rate of HIV infection in youth is fueled by the increased number of street youth. Studies revealed that HIV sero-prevalence rates for street youth are 10–25 times higher than other groups of adolescents [4].

The United Nations report estimated that about 150 million youth are homeless or spent most of their time on the street. The problem is becoming a worldwide phenomenon and majority of them live in large cities and urban areas of developing countries. Street youths are among the high risk, insecure and vulnerable groups to various forms of abuses and diseases. They are particularly exposed to HIV/AIDS [5,6].

Urbanization, poverty, lack of family support, peer influences and absence of employment, which are common in developing countries, are the leading causes to street life. This increasing number of street youth is one of the most serious problems in Ethiopia [7]. According to UNICEF report, there are approximately 500,000-700,000 street youth nationally [8]. Many of these street youth are found in streets of major towns like Gondar. They lives in conditions of sever deprivation which place them at all kind of health risks [9].

High risk sexual activities include unprotected sex, sex before legal consent and multiple sexual partners put youth at risk for teen pregnancy and sexual transmitted diseases. However, Risky sexual behaviors of street youth and its predictors in Gondar town have never been studied before. Therefore, this study was conducted to assess the prevalence of HIV/AIDS risky sexual behaviors and its predictors among street youths in Gondar town, Northwest Ethiopia.

## Methods

A community based cross sectional study was conducted from March to April, 2011 in Gondar town. It is located about 750 km away from Northwest of Addis Ababa, the capital of Ethiopia. According to the 2007 census projection, the town has a total population of 227,100 [10]. Many young and poor Ethiopians come to the town from the nearby districts and live in the streets as beggars and shoe shiner.

The participants of this study were street youth aged 10–24 who have lived in Gondar town for at least six months. The definition of street youth in this study was taken from WHO’s training module on substance use, sexual and reproductive health including HIV/AIDS and STDs. Street youth are defined as young people: who are ‘of the street’, having no home but the streets. Their family may have abandoned them or they may have no family member left alive. While who are ‘on the street’, are defined as those who visit their family regularly. They might even return every night to sleep at home, but spends most days and some nights on the street because of poverty, overcrowding, sexual or physical abuse at home [11]. For the purpose of this study, censes was first conducted to estimate the approximate number of street youth and identify their common place of residence. All the 467 street youth who live in Gondar town for at least six month on the street were included in this study.

Socio demographic data; sex, age, occupation, religion, former residence, educational level, occupation, and duration of stay on the street were collected. HIV/AIDS risky sexual behaviors of street youth were assessed using questionnaire adopted from Behavioral Surveillance Survey and other published articles which was modified to be applicable for the local context. Data were collected via interview using structured questionnaire which was first prepared in English and then translated to the local language Amharic. Pretest was done out of the study area and modifications were done accordingly. Data collectors were diploma nurses working at different health institutions of Gondar town. Training was given to the data collectors for two days on the objective, confidentiality of information, respondents` right and techniques of interview prior to data collection. During data collection, frequent checkups were made by the principal investigator to ensure the completeness and consistency of the data.

The returned questionnaires were checked for completeness by the investigators. The data were entered in to EPI INFO version 3.5.3 and analyzed by SPSS version 16. Descriptive analysis was done to summarize the data in relation to the different variables.

Variables having *P* value ≤ 0.2 in the bivariate analysis were entered into multiple logistic regression model to control the effect of confounding. Odds ratio with their 95% confidence intervals were calculated to measure associations, and statistical significance was set at *P* < 0.05.

Ethical clearance was obtained from the ethical review board of Institute of Public Health, University of Gondar. The purpose and importance of the study were explained to the participants. Data were collected after full informed verbal consent was obtained from each participant. Confidentiality of the information has been maintained throughout by excluding names in the questionnaire and keeping their privacy by interviewing alone. They were also informed that participation was on voluntary basis and they can withdraw at any time if they are not comfortable about the interview.

## Results

### Socio demographic characteristics of participants

A total of 467 youth were interviewed. Of them, 268(58.4%) were on the street and 76 (16.3%) were females. About half (51.4%) of the respondents were in the age group of 15–19 years. Almost all (97.9%) the study participants had some type of job on the street. Four hundred eight (89.5%) were earning less than twenty Ethiopian Birr per day (about one USA dollar) [Table 1].

**Table 1:** Socio demographic characteristics of street youth in Gondar town, Northwest Ethiopia 2011

**Variables**	**Number**	**Percent**
**Type of street life**		
On the street	268	58.4
Off the street	199	41.6
**Sex**		
Male	391	83.7
Female	76	16.3
**Ethnicity**		
Amhara	390	83.5
Tigray	77	16.5
**Age group**		
10–14	118	25.3
15–19	240	51.4
20-24	109	23.3
**Religion**		
Orthodox Christian	429	91.9
Muslim	12	2.6
Others	26	5.5
**Former residence**		
Urban	269	57.6
Rural	198	42.4
**Educational level**		
Illiterate	151	32.3
Read and write only	110	24
Primary school (1–8)	184	39
Secondary School (9–12)	22	4.7
*Occupation		
Shoe shining	40	11.6
Carrying items	285	61.0
peddling	53	11.3
Transferring message	123	26.3
Washing cars	90	19.3
Begging	30	6.4
Sex for money	61	13.0
**Average income per day**		
Less than 20 birr	418	89.5
Greater than 20 birr	49	10.7
**Duration on the street**		
Less than or equal to one year	56	12.0
Greater than one year	411	88.0

*due to multiple answers the percentage is greater than 100%.

### Prevalence of HIV/AIDS risky sexual behaviors

A total of 288 (61.7%) respondents had sexual intercourse in their life time. Among these, 264 (91.7%) had more than one lifetime sexual partner. Two hundred fifty two (87.5%) of them had sexual intercourse in the past twelve months. Three fourth of sexually active males (75%) had sexual intercourse with commercial sex workers in the past 12 months. Seventy four percent of sexually active youth had ever used condom. However, 80.5% of them used condom inconsistently in the last 12 months. The main reasons for not using were perceived reduction of sexual pleasure (34.8%), followed by negligence to use condom (26.2%) and too expensive to buy condom (12.3%) [Table 2].

**Table 2:** HIV/AIDS risky sexual behaviors of street youth in Gondar town, Northwest Ethiopia, 2011

**Variables**	**Frequency**	**Percent**
**Ever had sexual intercourse n=467**	288	61.7
Yes	179	38.3
No		
*Reason to have sex n=288		
Personal desire	103	22
Peer pressure	38	8
Fell in love	54	11.5
Influence of chat or alcohol	32	6.8
Raped	13	2.7
Marriage	38	8.1
To get money	78	16.7
Others	2	0.4
**Lifetime number of sexual partner n=288**		
One	24	8.3
More than one	264	91.7
**Sexual intercourse in the last 12 months n=288**		
Yes	252	87.5
No	36	12.5
**Number of sexual partner in the last 12 months n=252**		
One	70	27.8
More than one	182	72.2
**Ever use of condom n=288**		
Yes	213	74
No	75	26
**Consistency condom use in the last 12 months n=213**		
Consistent	41	19.5
Inconsistent	172	80.5
**Reason for not to use condom in the last 12 months**		
Not available	9	3.7
Too expensive	30	12.3
Ashamed to ask my partner	13	5.3
Partner objected	15	6.1
Donot like it	3	1.2
Reduce sexual satisfaction	85	34.8
Ashamed to buy	8	3.3
Negligence	64	26.2
Others	17	7.0
**Condom use during last sexual intercourse n=213**		
Yes	103	48.3
No	110	51.7
**Sex with CSW in the last 12 months n=236**		
Yes	177	75
No	59	25

*the percentage is greater than 100% because of multiple answers.

### Factors associated with HIV/AIDS risky sexual behaviors

In the bivariate analysis, more than a year stay on the street and khat chewing were factors associated with having multiple sexual partners. In the multivariate analysis, Khat chewers were at higher risk of having multiple sexual partners than non chewers [AOR 6.509; 95% CI: 2.494, 16.989] [Table 3].

**Table 3:** Factors associated with having multiple sexual partners among street youth in Gondar town, Northwest Ethiopia, 2011

**Variable**	**Multiple sexual partner**			
	**Yes**	**No**	**COR (95% CI)**	**AOR (95% CI)**
Type of street life				
On the street type	124	7	1.00	1.00
Off the street types	140	17	0.465 (0.187, 1.158)	0.529 (0.159, 1.758)
HIV knowledge				
Comprehensive	46	1	1.00	1.00
Not comprehensive	218	23	2.206 (0.639, 36.85)	4.236 (0.518, 34.624)
Average income per day				
≤20 birr	223	22	1.00	1.00
>20 birr	41	2	2.022 (0.458, 8.932)	2.567 (0.489, 13483)
Former residence				
Urban	152	15	1.00	1.00
Rural	112	9	1.228 (0.519, 2.907)	1.367 (0.505, 3.698)
Duration on the street				
≤1 year	100	4	1.00	1.00
>1 year	164	20	0.328 (0.109, 0.987)	0.228 (0.045, 1.17)
Alcohol drinking				
Never	14	2	1.00	1.00
Yes	250	22	1.623 (0.347, 7.606)	0.565 (0.104, 3.061)
Khat chewing				
Never	43	12	1.00	1.00
Yes	221	12	5.140 (2.166, 12.198)	6.509 (2.494, 16.989)
Condom use practice				
Consistent	39	5	1.00	1.00
Inconsistent	225	19	1.518 (0.535, 4.305)	3.278 (0.762, 14.099)

Similarly, factors associated with inconsistent condom use were investigated using logistic regression. Those who stayed on the street for more than a year were more likely to have history of inconsistent condom use than those who stayed on the street for less than a year (AOR = 4.365, 95% CI; 1.801, 10.582). Moreover, those who came from rural areas were more likely to have inconsistent condom use as compared to those who came from urban [AOR = 10.961, 95% CI: 3.206, 37.473]. However, type of street life and khat chewing were not significantly associated with inconsistence condom use [Table 4].

**Table 4:** Factors associated with condom use among street youth in Gondar town, Northwest Ethiopia, 2011

	**Inconsistent**			
**Variables**	**Condom use practice**			
	**Yes**	**No**	**COR (95% CI)**	**AOR (95% CI)**
Type of street life				
On the street types	99	32	1.00	1.00
Off the street types	145	12	3.906 (1.918, 7.952)	1.626 (0.662, 3.998)
Former residency				
Urban	126	41	1.00	1.00
Rural	118	3	12.799 (3.859, 7.952)	10.961 (3.206, 37.473)
Income per day				
≤20 birr	211	211	1.00	1.00
>20 birr	33	10	532 (0.240, 1.177)	0.386 (0.146, 1.021)
Duration on the street				
≤1 year	73	31	1.00	1.00
>1 year	171	13	5.586 (2.765, 11.285)	4.365 (1.801, 10.582)
Multiple sexual partners				
No	19	5	1.00	1.00
Yes	225	225	1.518 (0.535, 4.305)	1.733 (0.471, 6.375)
Khat chewing				
Never	41	14	1.00	1.00
Yes	203	30	2.311 (1.127, 4.736)	1.973 (0.82,4.745)
HIVknowledge				
Comprehensive	201	40	1.00	1.00
Not comprehensive	43	4	2.139 (0.727, 6.295)	3.454 (0.051, 11.343)

## Discussion

This study provided the opportunity to look at HIV/AIDS related risk sexual behaviors among street youth in Gondar town, Northwest Ethiopia. It is found that out of the total street youth, 61.7% have ever had sexual initiation. Lack of psycho social support from parents, peer pressure, higher prevalence of substance abuse and being idle might be the possible reasons for this high prevalence of sexual initiation.

Among sexually active participants, high proportion (91.7%) of respondents reported that they had multiple sexual partners which is high when compared with previous study done in Dessie town (80.6%) [12]. This might be due to Gondar town is one of the tourist areas in Ethiopia that pose risk for having multiple sexual partners. The other reason could be the ten years time gap between the two studies. The life time prevalence of having multiple sexual partners was also high. Unstable life styles, higher level of exposure to sexual exploitation and sexual practice for means of survival due to economical problems can be the possible explanations.

Of sexually active youth, 74% had ever used condom and this was similar with a study conducted on street youth in Dessie town (73.8%) [12]. Inconsistent condom use put youth at a higher risk for HIV/AIDS and other STIs. Majority of the respondents (80.5%) were found to have history of inconsistent condom use. This might be due to lack of knowledge about condom use. Consistent with a study done in Ethiopia [13], Khat chewing was significantly associated with having multiple sexual partners. This may be a result of the physiological effects of khat and its active element [14]. Amphetamine and amphetamine-like substances such as khat bring about negative physical and physiological effects by causing the continual release of neurotransmitters, like norepinephrine and particularly dopamine. Norepinephrine increased sympathethic nervous system activity, which alerts the system of the body to react instinctively to emergency situations and to have a role in initiating sex drive. Again the association between khat chewing and multiple sexual partners may be explained through social, physiological or individual mechanisms [15-17]. A study showed that khat chewers are also alcohol drunker [13]. Alcohol may reduce judgment and behavior via physiological or socially learned mechanisms. In addition to this, a youth's personal characteristics or social environment by itself may lead to risky sexual activity and initiation of new sexual partnership [18].

Duration of stay on the street had influence on inconsistent condom use. Participants who stayed on the street for more than a year were more likely to have history of inconsistent condom use. This finding is important for the planning of strategies related to the reduction of inconsistent condom use that are important to avoid unintended pregnancies and STDs including HIV AIDS. Similarly, another study done in Dessie town reported significant association between duration of stay and inconsistent condon use. In the present study, the use of condom inconsistently at last sexual relation was also much more frequent among youth who came from rural areas. The high rate of inconsistent condom use in this study was an indication of the fact that high risk sexual behaviors were widely practiced by the study group in Gondar town. This calls for a well organized information, education and communication effort to bring about behavioral change.

This study has the following strengths: the study has focused on marginalized and neglected group of people who are highly vulnerable to HIV and other STIs where adequate information and studies are lacking. This might certainly fill some of the knowledge gaps and serve as a base line information for future studies. The questionnaire used was adopted partly from the Behavioral Surveillance Survey tool (BSS) and from the previously published papers. In addition, high response rate was the other strength of this study. However, the difficulty to discuss sexual matter in face to face interview might bring social desirability bias though the survey was done anonymously by arranging same sex interviewer. Underreporting may have occurred, as some questions were of a sensitive nature.

## Conclusion

This study finding showed that high number of street youth had multiple sexual partners and the practice of inconsistent condom use was very high. Khat chewing was found to be significantly associated with having multiple sexual partners. Duration of stay on the street and former residence out of Gondar town were factors significantly associated with inconsistent use of condoms. Information education communication or behavioral change communication campaigns and reducing the duration of stay on the street are important recommendations to reduce the occurrence of risky sexual behaviors among street youth.

## Competing interests

The authors declare that they have no competing interests.

## Authors’ contributions

NT, ZB, TA, and KA participated in all steps of the study from its commencement to write up. They have reviewed and approved the submission of the manuscript.
